# Patient Endothelial Colony-Forming Cells to Model Coronary Artery Disease Susceptibility and Unravel the Role of Dysregulated Mitochondrial Redox Signalling

**DOI:** 10.3390/antiox10101547

**Published:** 2021-09-29

**Authors:** Marie Besnier, Meghan Finemore, Christine Yu, Katharine A. Kott, Stephen T. Vernon, Nicole A. Seebacher, Elijah Genetzakis, Anamarija Furman, Owen Tang, Ryan L. Davis, Thomas Hansen, Peter J. Psaltis, Kristen J. Bubb, Steven G. Wise, Stuart M. Grieve, Belinda A. Di Bartolo, Gemma A. Figtree

**Affiliations:** 1 Cardiovascular Discovery Group, Kolling Institute, University of Sydney, Sydney, NSW 2006, Australia; marie.besnier@sydney.edu.au (M.B.); meghan.finemore@sydney.edu.au (M.F.); Christine.yu1@health.nsw.gov.au (C.Y.); katharine.kott@sydney.edu.au (K.A.K.); sver4267@uni.sydney.edu.au (S.T.V.); elijah.genetzakis@sydney.edu.au (E.G.); anamarija.furman@sydney.edu.au (A.F.); owen.tang@sydney.edu.au (O.T.); Tommi_hansen1@hotmail.com (T.H.); kristen.bubb@monash.edu (K.J.B.); belinda.dibartolo@sydney.edu.au (B.A.D.B.); 2 Department of Cardiology, Royal North Shore Hospital, Sydney, NSW 2065, Australia; 3 Department of Oncology, University of Oxford, Oxford OX1 2JD, UK; nsee6009@uni.sydney.edu.au; 4 Neurogenetics Group, Kolling Institute, St Leonards, NSW 2065, Australia; ryan.davis@sydney.edu.au; 5 Faculty of Medicine and Health, University of Sydney, Sydney, NSW 2006, Australia; stuart.michael.grieve@gmail.com; 6 South Australian Health and Medical Research Institute, Adelaide, SA 5000, Australia; peter.psaltis@sahmri.com; 7 Adelaide Medical School, University of Adelaide, Adelaide, SA 5005, Australia; 8 Biomedicine Discovery Institute, Monash University, Clayton, VIC 3800, Australia; 9 School of Medical Sciences, Chronic Diseases Theme, University of Sydney, Sydngy, NSW 2006, Australia; steven.wise@sydney.edu.au; 10 Charles Perkins Centre, University of Sydney, Sydney, NSW 2006, Australia; 11 Charles Perkins Centre, Sydney Translational Imaging Laboratory, University of Sydney, Sydney, NSW 2006, Australia; 12 Department of Radiology, Royal Prince Alfred Hospital, Camperdown, Sydney, NSW 2050, Australia; 13 Cardiothoracic and Vascular Health, Kolling Institute of Medical Research, 10 Westbourne Street, St Leonards, NSW 2065, Australia

**Keywords:** coronary artery disease, biomarkers, endothelial cells, risk factors, atherosclerosis

## Abstract

Mechanisms involved in the individual susceptibility to atherosclerotic coronary artery disease (CAD) beyond traditional risk factors are poorly understood. Here, we describe the utility of cultured patient-derived endothelial colony-forming cells (ECFCs) in examining novel mechanisms of CAD susceptibility, particularly the role of dysregulated redox signalling. ECFCs were selectively cultured from peripheral blood mononuclear cells from 828 patients from the BioHEART-CT cohort, each with corresponding demographic, clinical and CT coronary angiographic imaging data. Spontaneous growth occurred in 178 (21.5%) patients and was more common in patients with hypertension (OR 1.45 (95% CI 1.03–2.02), *p* = 0.031), and less likely in patients with obesity (OR 0.62 [95% CI 0.40–0.95], *p* = 0.027) or obstructive CAD (stenosis > 50%) (OR 0.60 [95% CI 0.38–0.95], *p* = 0.027). ECFCs from patients with CAD had higher mitochondrial production of superoxide (O_2_^−^–MitoSOX assay). The latter was strongly correlated with the severity of CAD as measured by either coronary artery calcium score (R^2^ = 0.46; *p* = 0.0051) or Gensini Score (R^2^ = 0.67; *p* = 0.0002). Patient-derived ECFCs were successfully cultured in 3D culture pulsatile mini-vessels. Patient-derived ECFCs can provide a novel resource for discovering mechanisms of CAD disease susceptibility, particularly in relation to mitochondrial redox signalling.

## 1. Background

The key role of the Standard Modifiable cardiovascular Risk Factors (SMuRFs: hypertension, cholesterol, diabetes mellitus and smoking) in driving the development and progression of coronary artery disease (CAD) has been well-recognised at a population level and has been the target of successful primary prevention strategies for over 50 years. However, we have previously reported that up to 27% of ST elevation myocardial infarction (STEMI) patients, presenting with life-threatening events, did not actually present with any SMuRFs [1]. Additionally, in 62,048 STEMI patients from the SWEDEHEART registry, we found that these “SMuRFless” patients suffered a 47% higher 30-day-mortality than their counterparts with standard risk factors [2]. Moreover, a proportion of patients with CAD progress rapidly to have recurrent heart attacks despite optimal medical management, reflecting residual risk and susceptibility [3]. While these data highlight the variety of individual host responses responsible for patient susceptibility or resilience to the development of CAD, there is currently a paucity of biomarkers that reflect the actual amount of disease present at the level of the arterial wall. Overall, this body of work highlights the common misconception that STEMI only occurs in patients with SMuRFs and emphasises the need to better understand the pathophysiology of atherosclerosis and the necessity to discover novel biomarkers that might point to mechanisms of an individual’s disease susceptibility and be useful for predicting and managing risk.

A major challenge in dissecting differences in molecular signalling that explains individual variation in atherosclerosis susceptibility is the pragmatic lack of access to diseased tissue. This is in stark contrast to the precision approach to cancer, where molecular characterisation of individual patient’s tumours drives the selection of therapies. A model of patient-derived arterial cells in culture that maintain specific signalling characteristics and biology from the in vivo state could be invaluable in the quest to unravel new susceptibility and resilience mechanisms for atherosclerosis and may offer the first steps towards personalised therapies. Selectively-cultured endothelial progenitor cells (EPCs), derived from a patient’s peripheral blood, may provide such an opportunity.

The concept of circulating EPCs emerged in 1997 when Asahara et al. isolated a CD34^+^ subpopulation of peripheral blood cells for the first time and showed their capacity to differentiate into endothelial cells and to contribute to post-ischaemic neovascularisation in vivo [4]. Since then, two types of EPCs have been distinguished based on the time of appearance since isolation: the early outgrowth EPCs (within 10 days) and the late outgrowth EPCs (often between 1–3 weeks). The latter are now considered to be “true” EPCs [5]. The origin of the cells remains unclear. While, for some time, they were believed to be of bone marrow origin [6], more recent studies suggested that they stem from a tissue vascular niche instead [7]. For years, different names have been used to identify these cells—late outgrowth EPCs, endothelial colony-forming cells (ECFCs) or blood outgrowth endothelial cells (BOEC)—however it has recently been suggested that the label ECFC should be adopted to harmonise the literature [8], and we will use this term henceforth.

ECFCs have a high proliferative capacity, express endothelial cell markers and are phenotypically and functionally similar to mature endothelial cells [9,10,11,12,13], making them a potential tool for modelling an individual’s endothelial phenotype [5]. To date, they have been showed to have an altered function in various conditions and diseases ranging from diabetes [14], chronic lung disease [15] to the COVID-19 infection [16]. Here, we examine the feasibility and validity of using patient-derived ECFCs for discovering novel mechanisms of susceptibility and resilience to atherosclerosis, applied to a large, well-phenotyped cohort of individuals in the BioHEART-CT cohort [17]. This cohort of patients had advanced CT coronary angiography (CTCA) imaging of their coronary arteries, enabling precise disease quantification down to the subclinical level, granting the ability to identify a truly healthy control group. Here, we hypothesise that ECFCs can act as a model that reflects the endothelial function of the host, retaining a “memory” of their disease state, and provides a powerful resource for unravelling new mechanisms and biomarkers of coronary atherosclerosis.

## 2. Methods

### 2.1. Study Population

The BioHEART-CT study is a multi-centre, prospective cohort study designed to identify new biomarkers of atherosclerosis in patients with suspected CAD. BioHEART-CT is registered with the Australia and New Zealand Clinical Trials Network (ACTRN12618001322224) and the protocol has been described in detail previously [18,19]. In brief, stable patients were recruited at the time of clinically-indicated CTCA. Written informed consent was obtained, clinical and demographic data collected by questionnaire, peripheral venous blood samples collected, and CTCA imaging data acquired. The inclusion criteria were patients referred for investigation of suspected CAD who were over the age of 18, willing and able to provide informed consent and to participate in follow-up. Exclusion criteria included patients that were highly dependent on medical care who were unable to provide informed consent, and patients unwilling or unable to participate in ongoing follow-up. Additionally, patients with prior coronary artery bypass grafts or prior coronary artery stents were excluded from the cell function analysis as CTCA scoring using the Gensini system is not valid for this group. Patients included in this study were recruited between 2016 and 2019.

### 2.2. Definition of Risk Factors

Anthropometric parameters obtained included height, weight, and body mass index (BMI). Self-reported prior diagnosis of hypertension, hypercholesterolaemia, diabetes mellitus, current smoking (regularly smoked within the previous 12 months), or a smoking history of 10 or more pack-years (significant smoking history) were recorded. In addition to self-reported history, participants that were taking cholesterol lowering or anti-glycaemic pharmacotherapy at the time of recruitment were also considered to have hypercholesterolaemia or diabetes mellitus, respectively. Biochemical parameters, including fasting cholesterol profiles, fasting blood glucose or HbA1c were not available. A family history of ischaemic heart disease was considered significant if it was, in a first degree, relative under the age of 60 years.

### 2.3. Imaging Analysis

CTCAs were performed using a 256-slice CT scanner with standard clinical protocols applied [17,19]. If required, heart rate limiting medications (beta-blockers or ivabradine) were given orally for heart rate optimisation prior to the CTCA. Current recommendations [20] were followed to minimise radiation doses. Coronary artery calcium score (CACS) was assessed using vendor-specific software, utilising the Agatston method [21]. CTCAs were scored using the validated 17-segment Gensini score [22,23], which represents the total amount of calcified and non-calcified plaque present.

### 2.4. Biological Samples and ECFC Growth

Peripheral blood samples were collected following insertion of the peripheral venous cannula required for the CTCA. Blood was immediately transferred into lithium heparin pathology tubes and stored at room temperature. Peripheral blood mononuclear cells (PBMCs) were isolated within four hours of blood collection using a standard gradient-separation Ficoll preparation [24]. Briefly, PBMCs were freshly plated into 0.1% gelatine-coated flasks at a density of 2.5 × 10^4^ cells/cm^2^ in endothelial cell growth medium (EGM-2) containing 2% foetal bovine serum (EGM2 bulletkit, Lonza, Australia). The flasks were cultured in standard conditions (37 °C with 5% CO_2_) for up to 21 days, with regular monitoring for spontaneous growth of ECFCs.

### 2.5. Assessment of ECFCs Phenotype

Cell culture: ECFCs were maintained under standard conditions in EGM-2 basal medium (Lonza, Basel, Switzerland) supplemented with EGM-2 SingleQuot Kit (Lonza) containing supplements and growth factors according to recommendations, with a final foetal bovine serum (FBS) concentration of 2%. Cells used in this study were between passages 3 and 5.

Flow cytometry: For the characterization of the ECFCs, 10^5^ cells were incubated with human receptor Fc block (BioLegend, San Diego, CA, USA; 422301) for 10 min at room temperature. Cells were individually stained using a 1:20 dilution in staining buffer (BioLegend) of CD31-APC (BioLegend, #303115), CD34-PE (BioLegend, #343605), CD133-Brillant Violet 421 (BioLegend, #372807), and CD14-FITC (BioLegend, #367115) in a cell-staining medium (BioLegend, #420201) for 30 min on ice while being protected from light. Cells were washed and resuspended in staining buffer before being analysed on a BD LSRFortessa cell analyser (BD Science, Franklin Lakes, NJ, USA) flow cytometer. An unstained sample was used as a negative control. Quantification of the antigenic profile was performed using BD FACSDiva software.

Measurement of Nitric oxide: The intracellular nitric oxide (NO) level in ECFCs was measured using a NO reactive fluorescent dye, 4-Amino-5-Methylamino-2′,7′-Difluorofluoroscein Diacetate (DAF-FM). Cells were treated with 2.5 μM DAF-FM for 15 min before stimulation with acetylcholine (1 µM) for 15 min. Cells were then washed, fixed and mounted using mounting media (Thermofisher Scientific, Scoresby, Australia) with 4′,6-diamidino-2-phenylindole (DAPI). Cells were imaged using the 20× magnification objective of the EVOS FL Auto (AMAFD1000, ThermoFisher Scientific, Waltham, MA, USA).

Tube formation assay: 50 µL of Cultrex PathClear 3-D Culture Matrix reduced growth factor basement membrane extract (Matrigel^TM^, R&D Systems, Minneapolis, MN, USA) was added into a 96-well plate. Matrigel was then incubated at room temperature for 15 min, followed by a further 15 min incubation at 37 °C to allow the Matrigel to set. 1.5 × 10^4^ ECFCs from patients were added per well in duplicate. EGM-2 medium was added to wells up to 200 µL. Images were obtained after 6 h using 4× magnification on the EVOS FL Auto (AMAFD1000, ThermoFisher Scientific, Waltham, MA, USA), which was maintained at a temperature of 37 °C and 5% CO_2_ using carbogen. The number of branches per picture was counted using Image J. The tube formation was expressed as number of branches per initial number of cells plated at time 0 to account for seeding variability. The results are expressed as mean ± standard error of the mean (SEM).

Scratch assays: 2 × 10^4^ cells were seeded into a 96 well plate in triplicate and incubated overnight to reach 90–100% confluence. Cell media was removed, and a denuded zone was made with a 10 µL pipette tip. We added 200 µL of EGM-2 back into wells and images were obtained at 4× magnification using Evos FL Auto (AMAFD1000, ThermoFisher Scientific, MA, USA) at 0, 8 and 24 h. The scratch closure was analysed using Image J at the three time points and expressed as mean percentage of closure ± SEM.

Western blots: Cells in a T75 cm^2^ flask were washed with ice-cold phosphate buffered saline (PBS) twice, with 600 µL of lysis buffer containing 150 nmol/L NaCl (Sigma Aldrich, Burlington, MA, USA), 1% Igepal CA-630 (Sigma Aldrich, Burlington, MA, USA), and 50 mmol/L Trizma base pH 8.0 (Sigma Aldrich, MO, USA), which was supplemented by 1 tablet of PhosSTOP (Sigma Aldrich, MO, USA) and cOmplete ULTRA (Sigma Aldrich, MO, USA). Cells were mechanically removed via scraping, vortexed for 30 s and centrifuged at 20,000× *g* for 15 min at 4 °C. Protein concentration was determined using a MicroBCA Kit (Thermofisher scientific, Sydney, NSW, Australia) and 10 µg of protein lysate was denatured and run under reducing conditions on SDS-PAGE in 4–12% Tris-bis pre-cast gels (Thermofisher Scientific, Sydney, NSW, Australia). Gels were then transferred onto Immobilon polyvinylidene fluoride membrane (Sigma Aldrich, MO, USA). Membranes were incubated overnight at 4 °C in the following primary antibodies: ERK 1 + 2 (1:1000, #137F5, Cell Signalling Technology, Danvers, MA, USA); anti-phospho(Thr202/Tyr20)-ERK (1:2000, #4370S, Cell Signalling Technology, Danvers, MA, USA); anti-AKT (1:1000, #2938S, Cell Signalling Technology, Danvers, MA, USA); anti-phospho(Ser473)-AKT (1:1000, #9018S, Cell Signalling Technology, Danvers, MA, USA); anti-eNOS/NOS Type III (610297 BD Science, USA); NOX2 (1:1000, ab129068, Abcam, UK); NOX4 (1:5000, ab133303, Abcam, UK) and anti-actin (1:5000, #MAB1501, Merck). Secondary fluorescent antibodies specific to primary antibodies were then used (donkey anti-rabbit IRDye 680LT, LCR-926-32210 and goat anti-mouse 800CW, LCR-926-68023; Millenium Science, Mulgrave, Australia). Membranes were imaged using the Odyssey Imaging Platform (Licor, Lincoln, NE, USA) and band intensities were analysed using Image Studio software. Results are presented as mean ± SEM, relative to β-actin expression.

Mitochondrial reactive oxygen species production: The MitoSOX assay was performed according to the manufacturers protocol (Thermo Fisher Scientific, ThermoFisher, Waltham, MA, USA). Briefly, cells were seeded into 6-well plates, and allowed to grow for 24 h to 80% confluency. MitoSOX was added to a final concentration of 5 µM in HBSS (Mg^2+^, Ca^2+^) and incubated for 15 min at 37 °C in the dark. Cells were washed twice with HBSS (Mg^2+^, Ca^2+^), removed from culture plates with trypsin (Thermo Fischer Scientific) and further washed three times with HBSS (Mg^2+^, Ca^2+^). Cells were resuspended in 100 µL HBSS (Mg^2+^, Ca^2+^) for measurement at 510/580 nm by a LSRFortessa flow cytometer (BD). Antimycin A was used as a positive control (100 µM, 15 min/37 °C/dark; data not shown). An unstained control was also used to determine levels of background fluorescence and subtracted from all experimental readings. Triplicate experiments were carried out for all samples. For all samples, a minimum of 10,000 events were acquired. Data were exported for the analysis of the Mean Fluorescence Intensity (MFI) using FlowJo software (FlowJo, LLC, Ashland, OR, USA).

### 2.6. Culture of ECFCs in 3D Mini Vessels

LLC Scaffold production: Polycaprolactone (PCL) pellets (Sigma, 440744) and type A porcine-derived gelatin (Sigma, G2500) solution were prepared at 10% (*w*/*v*) concentration, by dissolving in HFP at a ratio of 90:10. The solution was left to homogenise overnight in a rotator at room temperature. PCL-gelatin grafts (2-mm inner diameter) were fabricated using standard electrospinning parameters of +20/−1 kV electrode voltages, 500 rpm mandrel rotation and a flow rate of 4 mL/h [25].

Cell culture: PCL-gelatin grafts were secured inside a resin printed bioreactor chamber, filled with EBM-2 medium. ECFCs at a cell density of 1 × 10^6^ were seeded into the graft lumen and allowed to attach for 1 h. Pulsatile flow was generated using a Welco WP10-P1/8 peristaltic roller pump. Pressure characterisation was performed using a PendoTech PREPS-N-012 in-line strain gauge pressure sensor. After 24 h, the graft was harvested from the bioreactor, and fixed with 10% formalin (Sigma, HT501128). For CD31 staining, the sample was first permeabilised in 0.1%Triton-X diluted in PBS and blocked with 5% BSA. The cells were then stained using AlexaFluor 488 conjugated anti-CD31 antibody (Abcam, ab215911) at 1:500 dilution with PBST and counter-stained with DAPI (ThermoFisher, R37605). The cells were then visualised under a fluorescent microscope at Ex/Em: 495/519 nm.

### 2.7. Statistical Analysis

Categorical variables are expressed as frequencies and percentages, continuous variables are expressed as means ± standard deviations if normally distributed, and medians with interquartile ranges if not normally distributed. Pearson’s Chi-squared test and Student’s *t* tests were used to compare categorical and continuous variables respectively. Univariate logistic regressions were performed to assess for association between the dependent variable, spontaneous ECFC growth, and prespecified independent variables: age, sex, obesity, hypertension, diabetes mellitus, hypercholesterolaemia, significant smoking history, current smoking, significant family history of CAD, statin, antiplatelet, anticoagulant, beta-blockers, angiotensin converting enzyme inhibitor (ACE)/angiotensin receptor blocker (ARB), presence of CAD (Gensini Score > 0), presence of calcified plaque (CACS > 0), and obstructive CAD (>50%). Multivariable logistic regression models were performed, adjusting for age, sex and independent variables with *p* < 0.1 for univariate associations. The association between MitoSOX superoxide production and both CACS and Gensini scores was demonstrated using bivariate linear regression analysis. Time course of migration assays was analysed using a mixed effect test on Graphpad Prism, version 8.4.3, San Diego, CA, USA. All other statistical analysis was performed in IBM SPSS Statistics, version 26, release 26.0.0.0, 64-bit edition, Sydney, NSW, Australia, with associated figures produced in Graphpad Prism, version 8.4.3., San Diego, CA, USA.

## 3. Results

### 3.1. Baseline Characterisation of ECFCs

**Figure 1:**
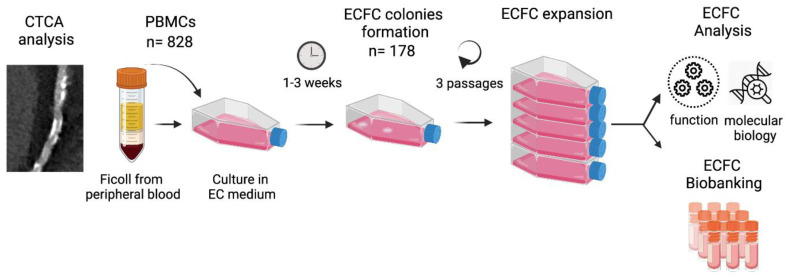
Schematic of the workflow of the study. CTCA: Computed Tomography Coronary Angiography, EC: Endothelial Cells, ECFC: Endothelial Colony Forming Cells, PBMCs: Peripheral Blood Mononuclear Cells. Created with BioRender.com (accessed on 15 September 2021).

**Figure 2:**
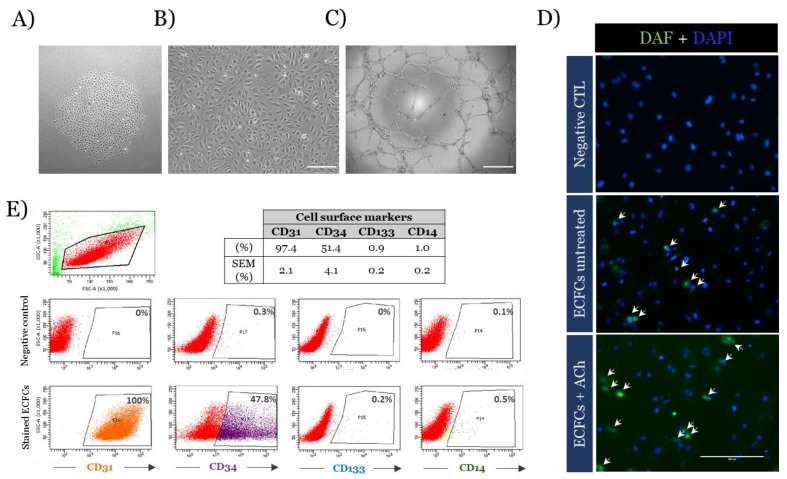
Characteristics of the patient derived ECFCs lines. ECFCs obtained from adult peripheral blood presented classical characteristics of ECFCs. (**A**) Bright-field images show an early colony of ECFCs and (**B**) a mature culture of ECFCs (passage 4). Scale bar = 200 µm. (**C**) Bright-field image of Matrigel tube formation after 6 h. Scale bar = 500 µm. (**D**) Fluorescent microscope images showing NO production of ECFCs in green using DAF-FM Diacetate (4-Amino-5-Methylamino-2′,7′-Difluorofluorescein Diacetate) and DAPI staining of cells treated or not with 1 µM of Acetylcholine (Ach) for 15 min. White arrows indicate cells positively stained. Cells that were not stained with DAF-FM were used as negative control. Scale bar = 200 µm. (**E**) Flow cytometry analysis for the characterisation of cell membrane markers of ECFCs. Representative pictures of the gating and a table showing the mean percentage of positive cell population ± SEM of *n* = 4 individual patient derived ECFC lines is provided.

A schematic flow chart is provided in Figure 1 to summarise the phases of growth. The phenotype of the ECFCs was consistent with their expected identity, the cells appearing as colonies between day 10 and 21 of culture (Figure 2A). After sub-culture, they formed a monolayer of cobblestone-shaped cells (Figure 2B) that were able to form tubes and branches on a Matrigel layer (Figure 2C) and produce NO (Figure 2D). Additionally, they expressed membrane markers of mature ECFCs [26] (Figure 2E, mean % ± SEM: CD31: 97% ± 2.1, CD34: ~51% ± 4.1, CD133: ~1% ± 0.2 expressed, CD14: ~1% ± 0.2, *n* = 4 different lines of ECFC).

### 3.2. Spontaneous Growth and Effect of Clinical Characteristics

**Figure 3:**
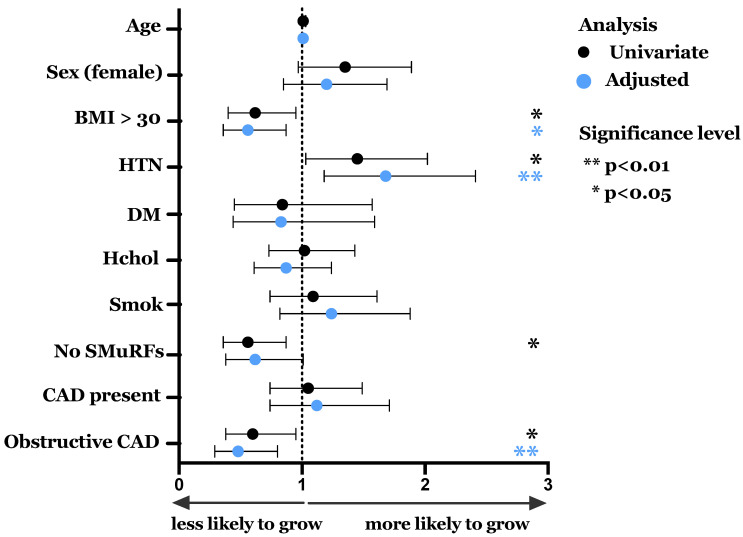
Odds ratios for association between clinical factors and successful ECFC Growth. Multivariable logistic regression models were performed, adjusting for age, sex and independent variables with *p* < 0.1 for univariate associations. Black dots represent univariate associations and blue dots represent adjusted odds ratios. Bars represent 95% confidence intervals. BMI: Body Mass Index > 30 kg/m^2^, HTN: hypertension, DM: Diabetes Mellitus; Hchol: Hypercholesterolemia. Smok: Significant smoking history (>10 pack years); No SMuRFs: >0 SMuRFs; CAD present: Gensini score > 0: Obstructive CAD (>50% stenosis).

We assessed associations between clinical and demographic characteristics associated with spontaneous ECFC growth—a key factor if the cells were to be considered a potential model to study mechanisms of disease susceptibility. In the 828 BioHEART patients studied, 178 (21.5%) had spontaneous growth of ECFCs. The clinical and demographic features of patients in the whole cohort and with successful growth of ECFCs are presented in Table 1. Some significant differences were seen between the groups in association with hypertension, obesity, the presence of any CAD, and the presence of obstructive (>0% stenosis) CAD. Figure 3 and Supplemental Tables S1 and S2 show unadjusted and adjusted odds ratios for spontaneous growth according to clinical and demographic variables. Patients with hypertension had a 45% higher rate of successful ECFC growth (OR 1.45 (95% CI 1.03–2.02), *p* = 0.031), an association that remained independent of age, sex, obesity and obstructive CAD in a multivariable model (adjusted OR 1.68 (95% CI 1.18–2.41), *p* = 0.010, Figure 3 and Supplemental Table S2). In contrast, a lower likelihood of growth was seen in patients with obesity (OR 0.62 [95% CI 0.40–0.95], *p* = 0.027; adjusted OR 0.56 [95% CI 0.36–0.87], *p* = 0.010) or obstructive CAD (OR 0.60 [95% CI 0.38–0.95], *p* = 0.027; adjusted OR 0.48 [95% CI 0.29–0.80], *p* = 0.004). An absence of SMuRFs was associated with a reduced likelihood of spontaneous growth (OR 0.56 [95% CI 0.36–0.87], *p* = 0.010). However, this association did not remain after adjustments for covariates. Spontaneous ECFC growth was not significantly associated with sex, cardiac medication or in any of the other major cardiac risk factors.

Despite the small differences in growth success of ECFCs in patients presenting with obesity, hypertension and obstructive CAD, the characteristics of patients with successful growth were clinically and demographically broad. Therefore, the cultured ECFCs could be used as a good representative material to study the endothelial function and molecular signature of patients with a wide range of conditions and risk factors.

When ECFC growth was successful, the appearance of the colonies occurred at 13.7 ± 4.5 days on average. To understand whether this parameter was affected by a patient’s characteristics, we investigated the association between the day of colony appearance and their clinical characteristics. There was no correlation between the time of colony appearance and CAD burden as assessed by the Gensini score (R^2^ = 0.0007; *p* = 0.77, Supplemental Figure S1). Similarly, there was no significant association between the days to colony appearance and any of the standard CAD risk factors, medications, or other CAD scores (Supplemental Table S3).

### 3.3. ECFC Phenotype Reflects the Coronary Artery Disease State of the Patient from Which They Were Derived

#### 3.3.1. Functional Imprint of Coronary Artery Disease

**Figure 4:**
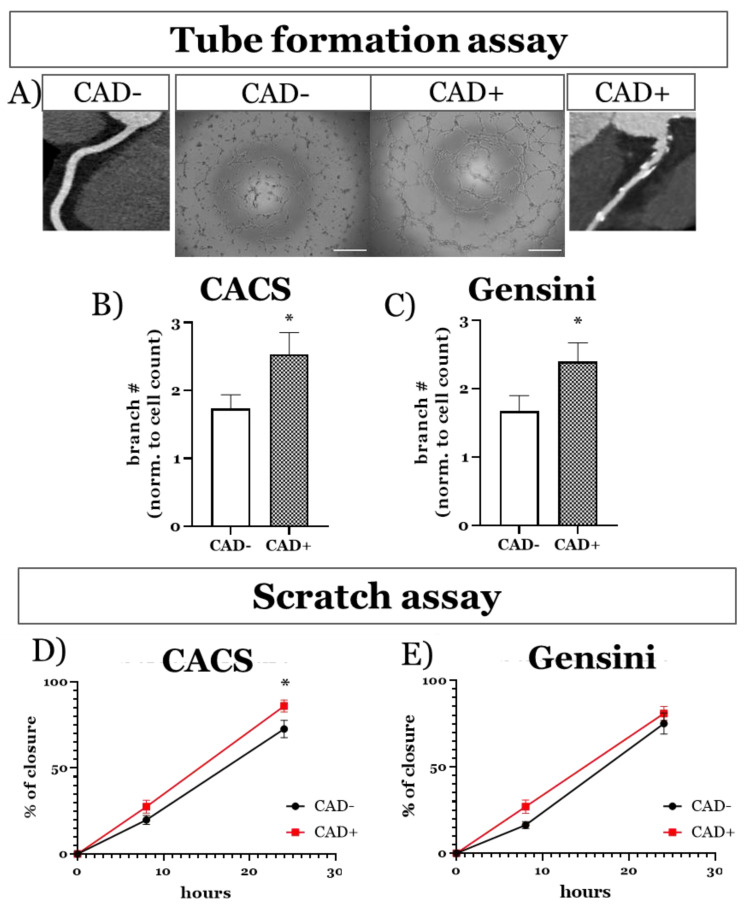
Proliferative and migration phenotype of ECFCs isolated from patients with and without CAD. (**A**) Reconstructions of coronary arteries from CTCA showing extensive atherosclerosis (CAD+, right) compared to a healthy, non-diseased vessel (CAD−, left) and representative bright-field images of matrigel tube formation assay performed with ECFCs from patients with (CAD+) and without (CAD−) CAD. Scale bar = 500 µm. (**B**,**C**) Bar graph of tube formation assay data of ECFCs from patients with or without CAD classified by either CACS or Gensini score respectively. Results are presented as mean ± SEM. Sample size: CACS = 0, *n* = 18; CACS > 0, *n* = 18; Gensini = 0, *n* = 13; Gensini > 0, *n* = 23. (**D**,**E**) Time course of closure in scratch assays on confluent cell monolayers in 96 well plates, showing the closure rates of ECFCs from patients with or without CAD classified by either CACS or Gensini score respectively. Results are presented as mean percentage of closure ± SEM. Sample size: CACS = 0, *n* = 9; CACS > 0, *n* = 15; Gensini = 0, *n* = 6; Gensini > 0, *n* = 18. * *p* < 0.05.

We next sought to investigate whether the function of ECFCs was affected when they were derived from patients with confirmed CAD. For this purpose, we used both the Gensini score and the CACS. ECFCs isolated from patients with CAD had a ≈45% increase in branch formation on Matrigel as compared to ECFCs of patients with no CAD (CACS > 0 vs. CACS = 0, *p* = 0.044 (Figure 4A,B); Gensini > 0 vs. Gensini = 0, *p* = 0.048 (Figure 4A,C)). Similarly, using a cell scratch assay, we found that the migratory and proliferative capacities of ECFCs from patients presenting CAD were ≈18% higher (CACS > 0 vs. CACS = 0, *p* = 0.03; Gensini > 0 vs. Gensini = 0, *p* = 0.18) (Figure 4D,E). The results of these assays after sex segregation are reported in Supplemental Figure S2A–D; no significant differences were identified.

#### 3.3.2. Molecular ECFC Phenotype Associated with Coronary Artery Disease Burden

We next examined whether ECFCs from patients with CAD had maintained a molecular imprint of the atherosclerotic disease present in the subject they were obtained from. To do so, we measured the expression of the NOX enzymes, known to be involved in oxidative stress and increased in coronary artery of patients with CAD [27]. Neither of the NOX2 or NOX4 isoforms were significantly changed in ECFCs isolated from patients with CAD compared to healthy controls (Table 2). Additionally, we did not find any significant differences in eNOS expression or activation of Akt or ERK1/2 that could explain the difference of function of ECFCs of patients with CAD (Table 2). The sex-segregation of the results are reported in Online Supplemental Tables S4 and S5.

**Figure 5:**
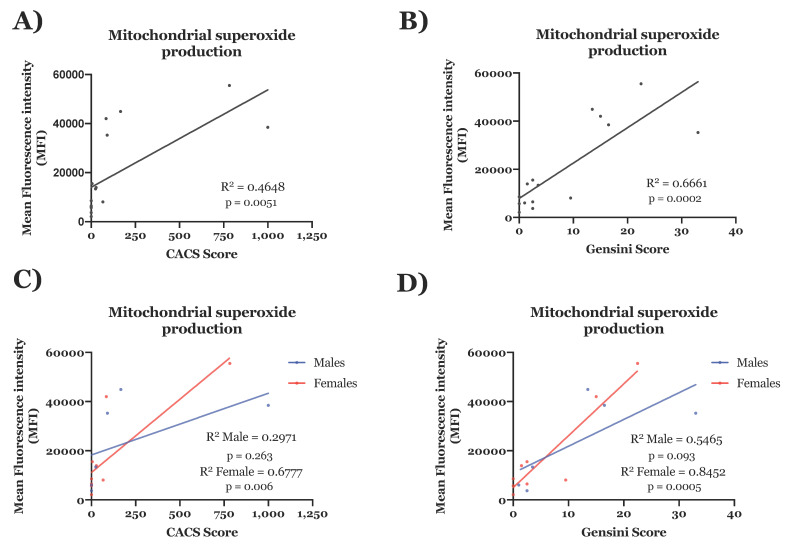
The mitochondrial production of superoxide is correlated to the severity of CAD. MitoSOX analyses were performed in ECFCs from patients with or without CAD to measure the mitochondrial production of superoxide. The presence of CAD was determined using CACS or Gensini score (**A**,**B**). Groups were split between females and males (**C**,**D**). Results are expressed as Mean Fluorescence Intensity (MFI). (**A**,**B**) show the results of both sexes of patients with or without CAD classified by either CACS or Gensini score respectively. (**C**,**D**) show the same results but sex segregated. Statistical correlations were derived using bivariate linear regression analysis. *n* = 15: male 6 and female 9.

Considering the importance of mitochondrial dysfunction and oxidative stress in the development of atherosclerotic plaques [28], we next assessed for associations between CAD and measures of mitochondrial function in the ECFCs. Using MitoSOX, we found that the mitochondrial production of superoxide of ECFCs was positively correlated with the severity of the CAD as measured by either the CACS (R^2^ = 0.46; *p* = 0.0051); Figure 5A) or the Gensini score (R^2^ = 0.67; *p* = 0.0002; Figure 5B). The correlation of MitoSOX was particularly strong in females, with an R^2^ of 0.84 in relation to the Gensini score (*p* = 0.0005; Figure 5D).

### 3.4. ECFCs Grown in 3D Mini Vessels

**Figure 6:**
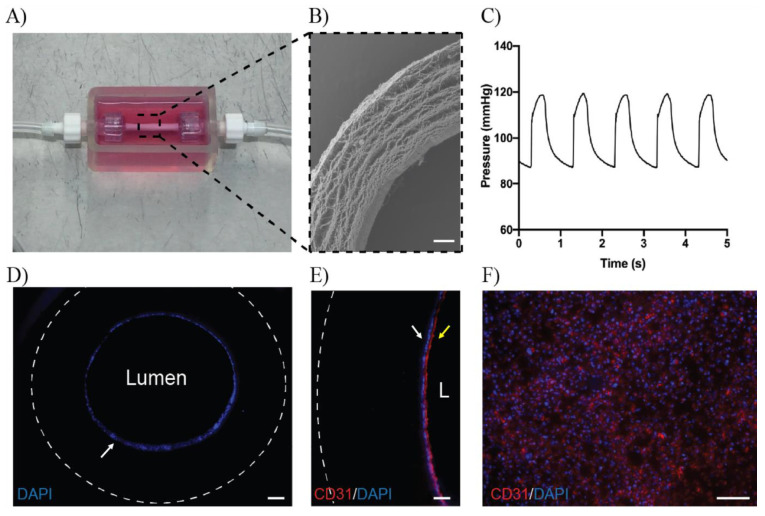
ECFCs rapidly form a confluent monolayer when grown in 3D under physiological conditions. (**A**) Vascular bioreactor Scheme 3. D cell growth on (**B**) fibrous PCL-gelatin scaffolds. Scale bar = 200 µm. (**C**) Seeded ECFCs were exposed to pulsatile pressure approximating human physiology. After 24 h, harvested grafts showed (**D**) a confluent monolayer of cells lining the graft lumen, staining positive for DAPI (White arrow, scale bar = 200 µm) and (**E**) CD31 (yellow arrow)/DAPI (white arrow). Scale bar = 50 µm. (**F**) Imaging of open graft sections supported the growth of a confluent layer of cells also staining CD31 positive. Scale bar = 100 µm.

To conclude, we examined the feasibility of growing patient derived ECFCs in a 3D culture environment more closely mimicking physiological conditions. The bioreactor was filled with media (Figure 6A) and an electrospun PCL-gelatin conduit fixed in place (Figure 6B). ECFCs seeded into the graft were allowed to attach to the conduit for 24 h in a 37C incubator, before pulsatile flow was applied to the system, approximating typical human conditions, with “pulse rates” of 60/min, and systolic/diastolic pressure equivalents of 120 mm Hg and 85–90 mm Hg diastolic (Figure 6C). At harvest, imaging of the graft cross-section showed a continuous layer of cells lining the lumen, staining positive for DAPI (Figure 6D) and CD31 (Figure 6E). Further imaging of open graft sections supported an evenly distributed layer of ECFCs staining positively for CD31 (Figure 6F).

## 4. Discussion

To our knowledge, this is the largest systematic study culturing and characterising the growth, function, and molecular signature of ECFCs derived from patients with well-characterised CAD. Whilst hypertension was associated with a higher rate of spontaneous growth, and obesity and obstructive CAD were associated with lower rates of spontaneous growth, these differences were relatively modest and did not preclude our ability to characterise cell signalling and function in these stratified patient groups. Indeed, diverse demographic and clinical features are well-represented in our bank of successfully-grown ECFCs. Importantly, ECFCs demonstrated at least a partial phenotypic imprint of their patient origin and CAD disease state, most distinctly demonstrated with dysregulated mitochondrial redox signalling. In addition, we have developed 3D cultured mini-vessels with patient-derived ECFCs to better mimic the physiology of the human endothelium [29]. These findings support the concept of the use of patient derived ECFCs as a feasible and relevant model for unravelling novel mechanisms of disease susceptibility and resilience.

Previous smaller studies have reported mixed findings regarding the association of CAD and risk factors with ECFC spontaneous growth and function. Vasa et al. reported that cardiovascular risk factors, including hypertension were associated with impaired endothelial progenitor function [30]. However, the literature has also reported conflicting results in numerous small studies [31]. Interestingly, in our study, hypertension was associated with increased spontaneous growth of ECFCs. In investigating whether physical forces on blood vessels could influence endothelial progenitors, Coppolino et al. found that stress stimulus induced by a cold pressor test in healthy and hypertensive patients could induce the release of circulating endothelial progenitors [32]. Therefore, one could hypothesize that the increase in vessel wall stress in patients with hypertension could be responsible for a higher number of circulating endothelial progenitors, and therefore lead to a better chance of growing ECFCs in patients with hypertension. Additionally, our data showed that the chance of successfully growing ECFCs from patients with obesity or obstructive CAD was decreased, independent of other covariates. We believe that the large size of our cohort and the CTCA-quantification of CAD burden provides stronger statistical power to more robustly demonstrate the effect of clinical and demographic characteristics on the overall growth success of ECFCs.

Regarding function, previous studies have reported that ECFCs derived from patients with CAD had reduced ability to form tubes and to migrate, suggesting a reduction of angiogenic capacities, compared with patients without a history of CAD [33,34]. Other studies have shown similar results to ours, identifying a slight increase of tube formation capacity of ECFCs isolated from CAD patients [35]. This difference in results may be partially explained by the methodology used to determine the presence or absence of CAD. Our CTCA scoring analysis enabled us to quantify the presence of any calcified disease using CACS, and additionally we were able to incorporate those with either calcified or non-calcified disease using the Gensini score. While our study used state-of-the-art CTCA techniques that allow visualisation of early coronary atherosclerosis and subsequent scoring, others have used either clinical events or cardiac catheterisation to define CAD, which is only sensitive for more extensive degrees of disease associated with significant narrowing of the artery. Most importantly in the current study, the non-CAD patients had a confirmed absence of visible CAD by CTCA imaging, while in most studies non-CAD or “healthy” patients are defined by the lack of a clinical event without coronary imaging being performed on them. Therefore, relying on events is associated with a significant risk of contamination of the “healthy” group with those who might have extensive non-obstructive and asymptomatic CAD.

The link between mitochondrial dysfunction, oxidative stress and atherosclerosis is well-established [28,36]. However, this is the first report of dysregulated mitochondrial redox status in patient derived ECFCs in association with atherosclerosis. The mechanisms for this dysregulation remain to be unravelled. The profoundly strong correlation of mitochondrial oxidative stress, as measured by MitoSOX, with quantitative measures of coronary atherosclerosis burden is of biological relevance.

The substantial dysregulation of mitochondrial redox status that is retained in cell cultures of ECFCs from patients with CAD has diagnostic and therapeutic potential. It will be important to perform further studies to understand whether this is upstream (involved in mechanisms) or downstream (reflecting disease activity) of the atherosclerotic disease process, or potentially both. Agents have been developed which are specific mitochondria-targeting antioxidants, including MitoQ [37]. In a mouse model of atherosclerosis and metabolic syndrome, MitoQ demonstrated benefits on macrophage content and cell proliferation within plaques but demonstrated no overall effect on plaque area over a 14-week period. There were additional benefits to hyperglycaemia and hepatic steatosis, as well as on multiple metabolically relevant lipid species. The usefulness of MitoQ treatment has not yet been investigated in patients with atherosclerosis, but it has recently been proven to significantly increase the endothelial function of aged persons and patients with peripheral artery disease [38,39], suggesting it has promising therapeutic potential.

As opposed to circulating molecular markers, patient-derived cells provide an opportunity for integrating candidate inflammatory, redox and mitochondrial markers, as well as applying unbiased approaches including genomic, transcriptomic, metabolomic and proteomic methods and to better align with biologically relevant groups stratified with the use of advanced imaging [40,41]. Our global hypothesis remains that major mechanisms for atherosclerosis are yet to be discovered and can be unravelled by comprehensive molecular characterisation of patient derived ECFCs. The findings of this study support the validity of this approach. The next steps will be to continue to expand the ECFC collection of the BioHEART study from the current 178 to 500 patient-derived ECFCs for detailed characterisation and as an open bioresource. We will apply state-of-the-art molecular phenotyping and cellular multi-omics to the patient-derived cells, and integrate this with deep clinical, imaging and outcome data using machine learning and advanced bioinformatic techniques. Unbiased systems biology approaches that integrate genomic activity measures (e.g., RNA, proteins, metabolites and DNA modifications) will be helpful to define disease-driving molecular processes as has been demonstrated for other complex vascular disease processes [42,43,44,45,46,47]. This will also be integrated with already-acquired “omics” data from plasma of these patients and analysed to build network models of relevant molecular processes, which will contribute to novel diagnostic and therapeutic opportunities, particularly relevant in patients with subclinical CAD where early effective preventative strategies can have profound benefits.

## 5. Conclusions

Considerable gaps remain in our understanding of individual susceptibility to coronary artery atherosclerosis, above and beyond traditional risk factors. A major challenge in unravelling missing biological mechanisms is the lack of accessible tissue or cellular material in large, well-phenotyped cohorts. Here, we have demonstrated the feasibility and potential of using patient-derived ECFC in both monoculture and 3D mini-vessels (Video S1) to explore mechanisms of susceptibility or resilience to CAD. This will be a valuable resource for both candidate and unbiased molecular and cellular discovery work, applying state-of-the-art systems approaches to identify novel mechanisms and markers of disease. Translation of these to new therapeutic targets and new diagnostic tools may help address major unmet needs in early detection and preventative strategies for patients at risk from CAD.

## Figures and Tables

**Table 1:** Baseline characteristics of the population of patients.

Characteristic	Whole Cohort (*n* = 828)	ECFC Growth (*n* = 178)	No ECFC Growth (*n* = 650)	*p* Value
Age, mean (SD)	60.9 (11.9)	62.1 (11.9)	60.6 (11.9)	0.14
Female, *n* (%)	379 (45.8)	92 (51.7)	287 (44.2)	0.07
Hypertension, *n* (%)	319 (38.5)	81 (45.5)	238 (36.7)	**0.03**
Diabetes mellitus, *n* (%)	69 (8.3)	13 (7.3)	56 (9.4)	0.58
Hypercholesterolaemia, *n* (%)	476 (57.5)	103 (57.9)	373 (57.4)	0.91
Significant smoking history, *n* (%)	190 (22.9)	43 (24.2)	147 (22.6)	0.67
Current smoker, *n* (%)	59 (7.1)	10 (5.6)	49 (7.5)	0.38
BMI, mean, (SD)	27.1 (5.0)	27.3 (5.1)	26.3 (4.8)	**0.02**
BMI >30 kg/m^2^, *n* (%)	191 (23.1)	30 (16.9)	161 (24.8)	**0.03**
Significant family history CAD, *n* (%)	163 (19.7)	29 (16.3)	134 (20.6)	0.20
SMuRFs—mean, (SD)	1.27 (0.96)	1.35 (0.87)	1.25 (0.98)	0.24
0 SMuRFs, *n* (%)	185 (22.3)	27 (15.2)	158 (24.3)	**<0.01**
Coronary artery calcium score—median, (IQR)	10 (0–177)	7.97 (0–140)	11 (0-193)	0.67
Calcified plaque present (CACS > 0)	494 (59.7)	109 (61.2)	385 (59.2)	0.63
Gensini score—median, IQR	4 (0–13)	3 (0–10)	4 (0–14)	0.27
CAD present (Gensini > 0)	547 (66.1)	119 (66.9)	428 (65.9)	0.80
Obstructive disease > 50% stenosis—*n*, (%)	165 (19.9)	25 (14.0)	140 (21.5)	**0.03**
Medication use:				
Anti-coagulant—*n*, (%)	76 (9.2)	18 (10.1)	58 (8.9)	0.63
Anti-platelet agent—*n*, (%)	145 (17.5)	32 (18.0)	113 (17.4)	0.85
Statin—*n*, (%)	282 (34.1)	66 (37.1)	216 (33.2)	0.34
Beta-blocker—*n*, (%)	122 (14.7)	21 (11.8)	101 (15.6)	0.21
ACE/ARB agent—*n*, (%)	256 (30.9)	63 (35.4)	193 (29.7)	0.15

Abbreviations: Standard Deviation (SD), Body Mass Index (BMI), Coronary Artery Disease (CAD), Interquartile Range (IQR), Standard Modifiable cardiovascular Risk Factors (SMURFs), Angiotensin Converting Enzyme (ACE), Angiotensin Receptor Blocker (ARB).

**Table 2:** ECFC Signalling Molecule Expression in Disease—CACS and the Gensini score.

Target (*n* CAD− vs. *n* CAD+)	CAD− (mean ± SEM)	CAD+ (mean ± SEM)	*p* Value
NOX2: - CACS (14 vs. 17) - Gensini (6 vs. 19)	1.00 ± 0.21 1.00 ± 0.26	0.93 ± 0.23 1.19 ± 0.23	0.92 0.68
NOX4: - CACS (11 vs. 14) - Gensini (6 vs. 19)	1.00 ± 0.27 1.00 ± 0.47	1.48 ± 0.36 1.26 ± 0.26	0.30 0.63
eNOS:- CACS (14 vs. 16) - Gensini (9 vs. 21)	1.00 ± 0.30 1.00 ± 0.34	1.34 ± 0.45 1.77 ± 0.49	0.54 0.34
AKT: - CACS (13 vs. 18) - Gensini (8 vs. 23)	1.00 ± 0.47 1.00 ± 0.60	0.93 ± 0.27 0.70 ± 0.18	0.90 0.53
pAKT: - CACS (13 vs. 18) - Gensini (8 vs. 23)	1.00 ± 0.17 1.00 ± 0.21	1.43 ± 0.37 1.09 ± 0.26	0.31 0.84
pAKT/AKT: - CACS (13 vs. 18) - Gensini (8 vs. 23)	1.00 ± 0.24 1.00 ± 0.30	0.66 ± 0.13 0.84 ± 0.16	0.16 0.62
ERK:- CACS (12 vs. 18) - Gensini (7 vs. 23)	1.00 ± 0.13 1.00 ± 0.18	1.36 ± 0.25 1.20 ± 0.19	0.28 0.60
pERK: - CACS (12 vs. 18) - Gensini (7 vs. 23)	1.00 ± 0.41 1.00 ± 0.63	1.82 ± 0.67 1.69 ± 0.55	0.31 0.52
pERK/ERK: - CACS (12 vs. 18) - Gensini (7 vs. 23)	1.00 ± 0.35 1.00 ± 0.50	1.26 ± 0.46 1.26 ± 0.39	0.69 0.73

## Data Availability

The data presented in this study are available on request from the corresponding author.

## Supplementary Materials

The following are available online at https://www.mdpi.com/article/10.3390/antiox10101547/s1, Figure S1: ECFC Days to Growth by Gensini Score; Figure S2: Sex segregation of ECFC function analysis in patients with or without CAD; Table S1: Univariate Associations with ECFC Growth; Table S2: Supplemental Table 2: Adjusted Associations with ECFC Growth—Adjusted for age, sex, obesity, hypertension and obstructive CAD; Table S3: Days to Colony Formation by Clinical Subgroups; Table S4: ECFC Signalling Molecule Expression in Disease—Calcium Score; Table S5: ECFC Signalling Molecule Expression in Disease—Gensini; Video S1: Video of patient-derived mini artery.
