# Supplementary material for: Patient Endothelial Colony-Forming Cells to Model Coronary Artery Disease Susceptibility and Unravel the Role of Dysregulated Mitochondrial Redox Signalling
Source: Antioxidants (Basel). 2021 Sep 29;10(10):1547. doi: 10.3390/antiox10101547 (PMC8532880; doi:10.3390/antiox10101547)
Supplement: Supplementary file 1 [file antioxidants-10-01547-s001.zip › Online supplement_ECFCpaper_130721.pdf]

Supplemental Figures

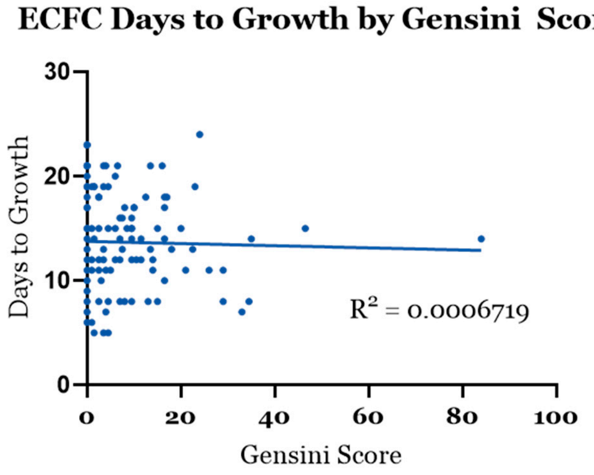

Supplement Figure S1: Scatterplot with linear regression line reflecting days until ECFC formation from adult peripheral blood mononuclear cells, demonstrating a lack of correlation with Gensini score. n=112.

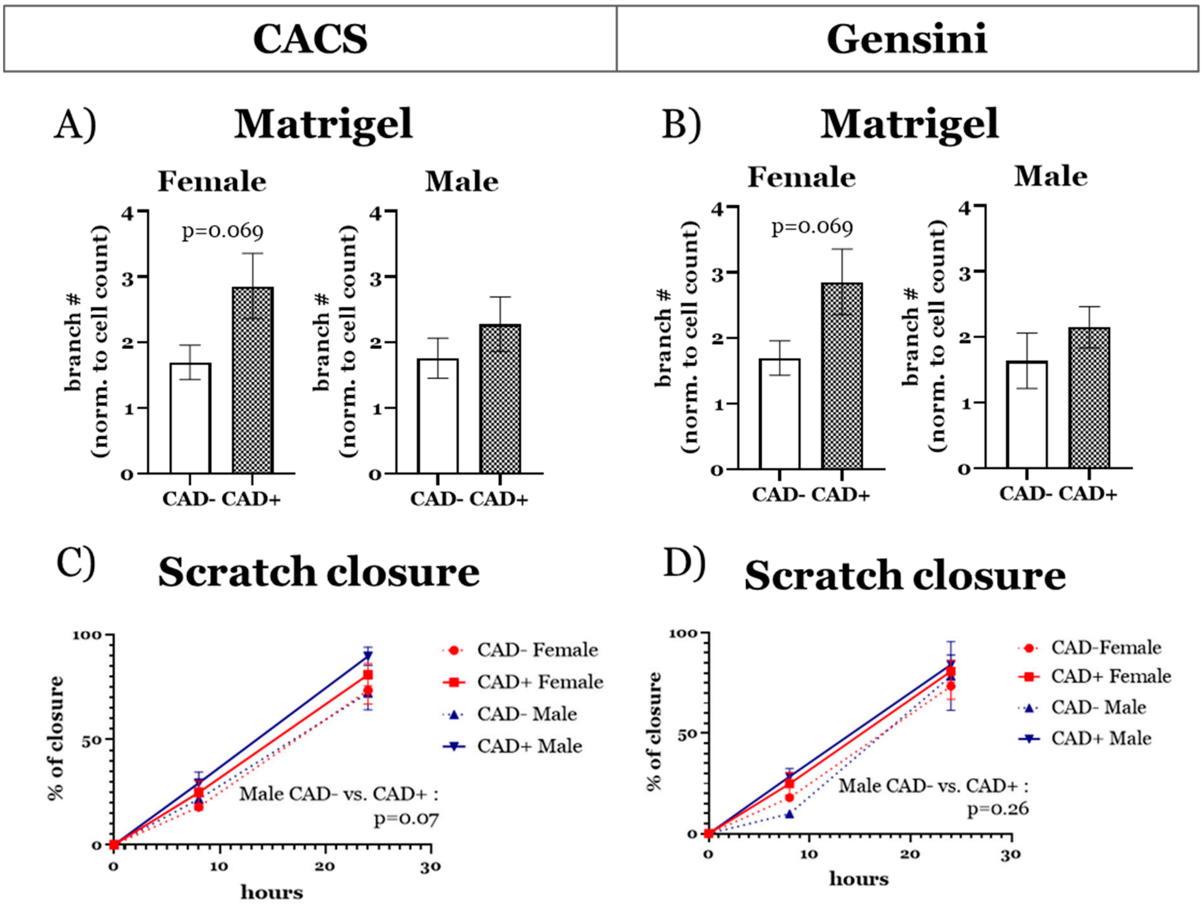

Supplement Figure S2: Sex segregation of ECFC function analysis in patients with or without CAD. Matrigel tube formation and scratch closure abilities were assessed in vitro on ECFCs from patients with CAD (CAD+) or not (CAD-). The presence of CAD was determined using CACS or Gensini score. Groups were split between females and males using biological sex difference. The sex segregated difference of tube formation of ECFCs from patients with or without CAD classified by either CACS (A) or Gensini score (B) is represented. Results are presented as mean ± SEM. Sample size: CACS=0, female = 7, male = 11; CACS>0, female = 8, male = 10; Gensini=0, female = 7, male = 6; Gensini>0, female = 8, male = 15. C-D) Scratch assays were performed on a confluent cell monolayer in 96 well plates. Results are presented as mean percentage of closure over time ± SEM. F and G) show the difference of closure abilities of ECFCs from patients with or without CAD classified by either CACS (C) or Gensini (D) score in a sex segregated way. Sample size: CACS=0, female = 4, male = 5; CACS>0, female = 6, male = 9 ; Gensini=0, female = 4, male = 2; Gensini>0, female = 6, male = 12.

Supplemental Tables

**Supplemental Table S1: Univariate Associations with ECFC Growth**			
**Variable**	**Odds Ratio**	**95% C.I.**	**p value**
Age	1.01	1.00 – 1.03	0.144
Female sex	1.35	0.97 – 1.89	0.074
Obese (BMI >30)	0.62	0.40 – 0.95	**0.027**
Hypertension	1.45	1.03 – 2.02	**0.031**
Diabetes Mellitus	0.84	0.45 – 1.57	0.575
Hypercholesterolaemia	1.02	0.73 – 1.43	0.909
Significant Smoking History CAD	1.09	0.74 – 1.61	0.665
Current Smoker	0.73	0.36 – 1.47	0.379
Significant Family History	0.75	0.48 – 1.17	0.200
No SMuRFs	0.56	0.36 – 0.87	**0.010**
Statin	1.18	0.84 – 1.67	0.337
Anti-platelet	1.04	0.67 – 1.61	0.854
Anti-coagulant	1.15	0.66 – 2.00	0.627
Beta-blocker	0.73	0.44 – 1.20	0.214
ACE/ARB	1.30	0.91 – 1.84	0.145
Calcified plaque present (CACS>0)	1.09	0.77 – 1.53	0.629
CAD present (Gensini>0)	1.05	0.74 – 1.49	0.801
Obstructive Disease (> 50% Stenosis)	0.60	0.38 – 0.95	**0.027**

**Supplemental Table S2: Adjusted Associations with ECFC Growth – Adjusted for age, sex, obesity, hypertension and obstructive CAD**			
**Variable**	**Odds Ratio**	**95% C.I.**	**p value**
Age	1.01	1.00 – 1.03	0.171
Female sex	1.20	0.85 – 1.69	0.308
Obese (BMI >30)	0.56	0.36 – 0.87	**0.010**
Hypertension	1.68	1.18 – 2.41	**0.004**
Diabetes Mellitus	0.83	0.44 – 1.59	0.579
Hypercholesterolaemia	0.87	0.61 – 1.24	0.451
Significant Smoking History CAD	1.24	0.82 – 1.88	0.301
Current Smoker	0.80	0.39 – 1.64	0.544
No SMuRFs	0.62	0.38 – 1.01	0.057
Significant Family History	0.74	0.47 – 1.16	0.183
Statin	1.07	0.74 – 1.54	0.731
Anti-platelet	0.96	0.62 – 1.50	0.865
Anti-coagulant	1.07	0.60 – 1.92	0.816
Beta-blocker	0.68	0.40 – 1.15	0.153
ACE/ARB	0.96	0.58 – 1.60	0.881
Calcified plaque present (CACS>0)	1.17	0.78 – 1.76	0.453
CAD present (Gensini>0)	1.12	0.74 – 1.71	0.587
Obstructive Disease (> 50% Stenosis)	0.48	0.29 – 0.80	**0.004**

Supplemental Table S3: Days to Colony Formation by Clinical Subgroups			
**Factor**	**Days - Factor Present**	**Days - Factor Absent**	**p value**
Sex – female	13.7 (4.4)	13.7 (4.5)	0.91
Obese (BMI > 30)	14.8 (4.3)	13.5 (4.5)	0.23
Hypertension	14.1 (4.8)	13.3 (4.2)	0.33
Diabetes Mellitus	14.5 (3.6)	13.6 (4.5)	0.55
Hyperlipidaemia	13.8 (4.5)	13.5 (4.5)	0.69
Current Smokers	11.7 (5.0)	13.8 (4.4)	0.26
Significant Smoking History	12.7 (4.3)	14.0 (4.5)	0.17
Significant Family History	13.3 (5.2)	13.8 (4.3)	0.65
Anti-coagulated	12.8 (4.2)	13.8 (4.5)	0.42
Taking anti-platelet agent	13.7 (4.3)	13.7 (4.5)	0.96
Taking statin	14.1 (3.8)	13.5 (4.8)	0.44
Takin beta-blocker	13.7 (5.0)	13.7 (4.4)	0.97
Taking ACE/ARB agent	13.9 (4.4)	13.3 (4.6)	0.58
CAD present (Gensini > 0)	13.5 (4.3)	14.2 (4.8)	0.37
Obstructive Disease > 50% Stenosis	13.8 (4.4)	13.7 (4.5)	0.90

Supplemental Table S4: ECFC Signalling Molecule Expression in Disease – Calcium Score			
**Target (n CAD- vs n CAD+)**	**CAD-** (mean ± sem)	**CAD+** (mean ± sem)	**p value**
NOX2 -Male (8 vs 9)<br>-Female (6 vs 8)	1.00 ± 0.27<br>1.00 ± 0.38	0.71 ± 0.21<br>1.27 ± 0.48	0.50<br>0.68
NOX4 -Male (6 vs 8)<br>-Female (5 vs 6)	1.00 ± 0.16<br>1.00 ± 0.61	1.56 ± 0.53<br>1.37 ± 0.52	0.34<br>0.65
eNOS -Male (8 vs 9)<br>-Female (6 vs 7)	1.00 ± 0.45<br>1.00 ± 0.39	1.36 ± 0.66<br>1.32 ± 0.62	0.67<br>0.68
AKT -Male (8 vs 9)<br>-Female (5 vs 9)	1.00 ± 0.56<br>1.00 ± 0.73	0.35 ± 0.19<br>2.80 ± 0.86	0.27<br>0.19
pAKT -Male (8 vs 9)<br>-Female (5 vs 9)	1.00 ± 0.25<br>1.00 ± 0.18	0.71 ± 0.21<br>2.38 ± 0.74	0.39<br>0.11
pAKT/AKT - Male (8 vs 9)<br>- Female (5 vs 9)	1.00 ± 0.22<br>1.00 ± 0.34	0.98 ± 0.26<br>0.44 ± 0.14	0.96<br>0.09
ERK -Male (7 vs 9)<br>-Female (5 vs 9)	1.00 ± 0.11<br>1.00 ± 0.27	1.37 ± 0.32<br>1.33 ± 0.40	0.35<br>0.58
pERK -Male (7 vs 9)<br>-Female (5 vs 9)	1.00 ± 0.43<br>1.00 ± 0.87	0.95 ± 0.83<br>2.73 ± 1.03	0.96<br>0.23
pERK/ERK -Male (7 vs 9)<br>-Female (5 vs 9)	1.00 ± 0.41<br>1.00 ± 0.71	0.44 ± 0.29<br>2.42 ± 0.97	0.27<br>0.22

Supplemental Table S5: ECFC Signalling Molecule Expression in Disease – Gensini			
**Target (n CAD- vs n CAD+)**	**CAD-** (mean ± sem)	**CAD+** (mean ± sem)	**p value**
NOX2 -Male (4 vs 13)<br>-Female (5 vs 9)	1.00 ± 0.29<br>1.00 ± 0.41	1.37 ± 0.31<br>1.10 ± 0.39	0.61<br>0.84
NOX4 -Male (2 vs 12)<br>-Female (4 vs 7)	1.00 ± 0.07<br>1.00 ± 0.77	1.27 ± 0.43<br>1.09 ± 0.50	0.81<br>0.75
eNOS -Male (4 vs 13)<br>-Female (5 vs 8)	1.00 ± 0.45<br>1.00 ± 0.41	3.62 ± 1.28<br>1.09 ± 0.50	0.29<br>0.90
AKT -Male (4 vs 13)<br>-Female (4 vs 10)	1.00 ± 0.79<br>1.00 ± 0.73	0.30 ± 0.67<br>2.06 ± 0.12	0.44<br>0.38
pAKT -Male (4 vs 13)<br>-Female (4 vs 10)	1.00 ± 0.34<br>1.00 ± 0.18	0.56 ± 0.12<br>2.02 ± 0.63	0.15<br>0.15
pAKT/AKT - Male (4 vs 13)<br>- Female (4 vs 10)	1.00 ± 0.38<br>1.00 ± 0.42	1.06 ± 0.21<br>0.74 ± 0.24	0.90<br>0.62
ERK -Male (3 vs 13)<br>-Female (4 vs 10)	1.00 ± 0.16<br>1.00 ± 0.22	1.37 ± 0.27<br>1.33 ± 0.31	0.32<br>0.95
pERK -Male (3 vs 13)<br>-Female (4 vs 10)	1.00 ± 0.82<br>1.00 ± 0.89	1.58 ± 0.91<br>2.00 ± 0.78	0.77<br>0.42
pERK/ERK -Male (3 vs 13)<br>-Female (4 vs 10)	1.00 ± 0.75<br>1.00 ± 0.78	0.74 ± 0.32<br>1.95 ± 0.78	0.73<br>0.49

Supplemental Legends for Video file

Video of patient-derived mini artery. ECFCs rapidly form a confluent monolayer when grown in 3D under physiological conditions. As described in the methods, a vascular bioreactor system facilitates 3D cell growth on fibrous PCL-gelatin scaffolds. Seeded ECFCs are exposed to pulsatile pressure approximating human physiology, at 37C, with arterial pressure of ~120 mm Hg systolic and ~90 mm Hg diastolic.
